# Esophageal submucosal hematoma during transnasal endoscopy: A rare case report

**DOI:** 10.1002/deo2.366

**Published:** 2024-04-15

**Authors:** Atsushi Kanamori, Yuji Nadatani, Nahoko Kushiyama, Akinobu Nakata, Akira Higashimori, Masaki Ominami, Tatsuo Kimura, Shinya Fukumoto, Yasuhiro Fujiwara, Toshio Watanabe

**Affiliations:** ^1^ Department of Premier Preventive Medicine/MedCity21 Osaka Metropolitan University Graduate School of Medicine Osaka Japan; ^2^ Department of Gastroenterology Osaka Metropolitan University Graduate School of Medicine Osaka Japan

**Keywords:** endoscopy, esophageal hematoma, esophageal intramural hematoma, esophageal submucosal hematoma, vomiting

## Abstract

Esophageal submucosal hematoma is a rare, often incidental complication of therapeutic endoscopic procedures marked by disrupted blood vessels beneath the esophageal mucosa, forming a hematoma. We report the unique case of a severely thin and alcoholic 38‐year‐old woman with a history of reflux esophagitis who developed an esophageal submucosal hematoma during an unsedated transnasal endoscopy for health check‐up. During the procedure, the patient experienced strong vomiting reflexes and vomited blood, leading to the initial suspicion of either Mallory‐Weiss syndrome or epistaxis. However, subsequent sedated endoscopy revealed an esophageal submucosal tumor‐like lesion and a mucosal laceration with blood clots, prompting a dual diagnosis of esophageal submucosal hematoma and Mallory‐Weiss syndrome. The bleeding was not severe enough to require hemostatic intervention. The patient opted for conservative treatment with vonoprazan, which resulted in the improvement and healing of the hematoma within 28 days. This is the first report of an esophageal submucosal hematoma during transnasal endoscopy and emphasizes the importance of including an esophageal submucosal hematoma and Mallory‐Weiss syndrome in the differential diagnosis of hematemesis encountered in similar scenarios. Factors such as severe thinness, daily alcohol consumption, and reflux esophagitis may have possibly contributed to the development of the esophageal submucosal hematoma in this patient.

## INTRODUCTION

In routine clinical practice, intravenous sedation is commonly used to alleviate discomfort during esophagogastroduodenoscopy (EGD) and is considered beneficial; however, its use is not recommended in routine health checkups in Japan as it poses the risk of cardiopulmonary complications.^1^

Transnasal endoscopy (TNE) using small‐caliber endoscopes (diameter less than 6 mm) that further helps reduce patient distress is gaining popularity in health checkups. TNE, being less invasive and uncomfortable than traditional oral endoscopy, is becoming the preferred method for patients and healthcare professionals in health checkups.^2^

Esophageal submucosal hematoma, a rare condition characterized by disrupted blood vessels beneath the esophageal mucosa leading to hematoma formation, is often an incidental complication of endoscopic procedures such as endoscopic injection sclerotherapy and endoscopic retrograde cholangiopancreatography.^3^, ^4^ However, its occurrence during TNE has not been documented. Therefore, we present a case of esophageal submucosal hematoma that occurred during TNE for screening purposes.

## CASE REPORT

**FIGURE 1:**
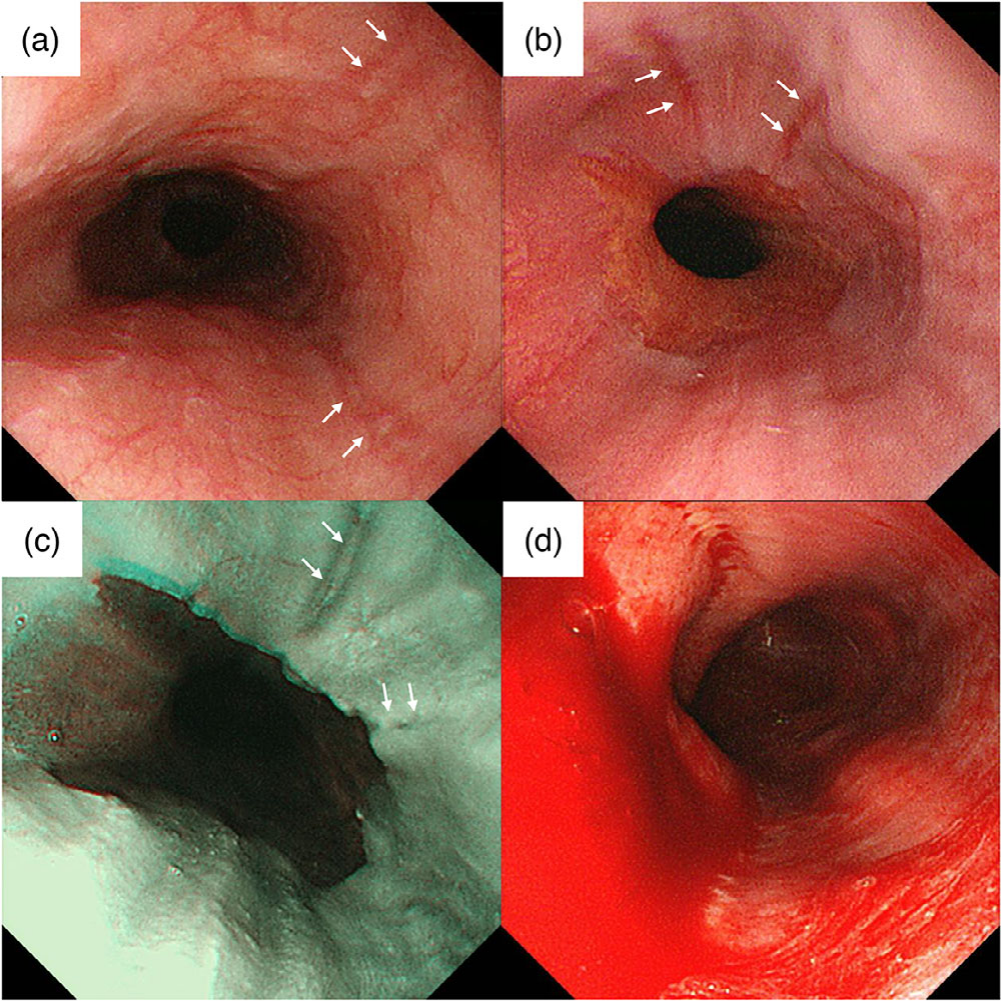
Endoscopic images of the esophagus taken previously and at present. (a) Several mucosal breaks are observed in the middle esophagus (white arrows). (b) Previously, several mucosal breaks >5 mm contiguous with the squamous‐columnar junction in the lower esophagus (white arrows). (c) The image shows mucosal breaks in the right to the anterior wall of the esophagus before hematemesis (white arrows). (d) After hematemesis, the source of bleeding is unknown because of massive blood retention in the esophagus.

A 38‐year‐old woman with a history of reflux esophagitis and successful eradication of a *Helicobacter pylori* infection visited our facility for a health checkup. The patient was not under any medication and reported a daily intake of 500 mL of beer for over 10 years, with no history of smoking or allergies. She was 159.8 cm tall and weighed 34.0 kg, having lost over 5 kg in the past 3 years, with a body mass index of 13.3 kg/m^2^. She reported increased stress and decreased food intake with no subjective symptoms. Recent imaging and blood tests revealed no tumors or metabolic diseases. The patient had undergone several unsedated TNE and had been diagnosed with reflux esophagitis (Los Angeles classification grade B) and esophageal hiatal hernia (Figure 1a,b) in the previous year. During the TNE procedures, the patient did not experience severe vomiting reflexes or tachypnea.

This year, TNE was performed as a health checkup for this patient. Pre‐endoscopic pretreatment was conducted by the Japanese guidelines and TNE was conducted by a board‐certified fellow with 9 years of experience without intravenous sedation using GIF‐XP290N (Olympus Medical System).

The patient experienced vomiting reflexes and tachypnea during the TNE, which improved by halting endoscopic movements and slow breathing. Mucosal breaks were noted at the esophagogastric junction (EGJ), similar to the findings of previous examinations (Figure 1c). Strong vomiting reflexes were triggered upon endoscopic insertion into the duodenum, and the patient vomited approximately 100 mL of blood. When the middle and lower esophagus were observed immediately after hematemesis, there was no influx of blood from the upper or middle esophagus. The initial suspicion was a Mallory‐Weiss syndrome, prompting an immediate examination of the EGJ and stomach; however, the bleeding source remained unidentified (Figure 1d).

**FIGURE 2:**
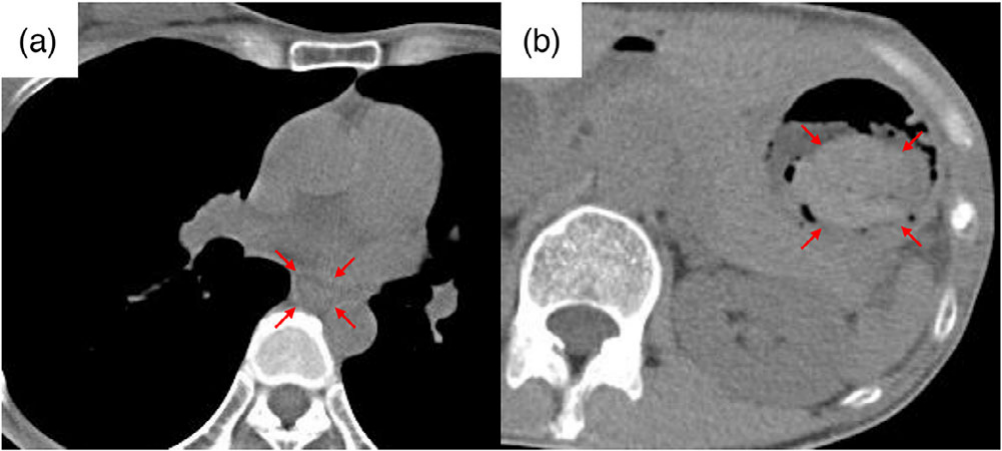
Plain computed tomography scan. (a) A continuous wall thickening from the middle to the lower thoracic esophagus was observed (red arrows). (b) A clearly demarcated hyperabsorptive zone, considered blood in the fundus of the stomach, was observed (red arrows).

The TNE had to be halted, and the patient was transferred to our affiliated university hospital since our center had no hemostatic equipment. She reported precordial pain but had normal vital signs. Blood tests showed a slight decrease in serum hemoglobin level from 13.0 to 12.3 g/dL; however, platelet count, prothrombin time, and activated partial thromboplastin time were all within normal ranges. Plain computed tomography revealed an edematous wall thickening from the middle thoracic esophagus to the EGJ and a suspected blood‐filled gastric reservoir (Figure 2a,b).

**FIGURE 3:**
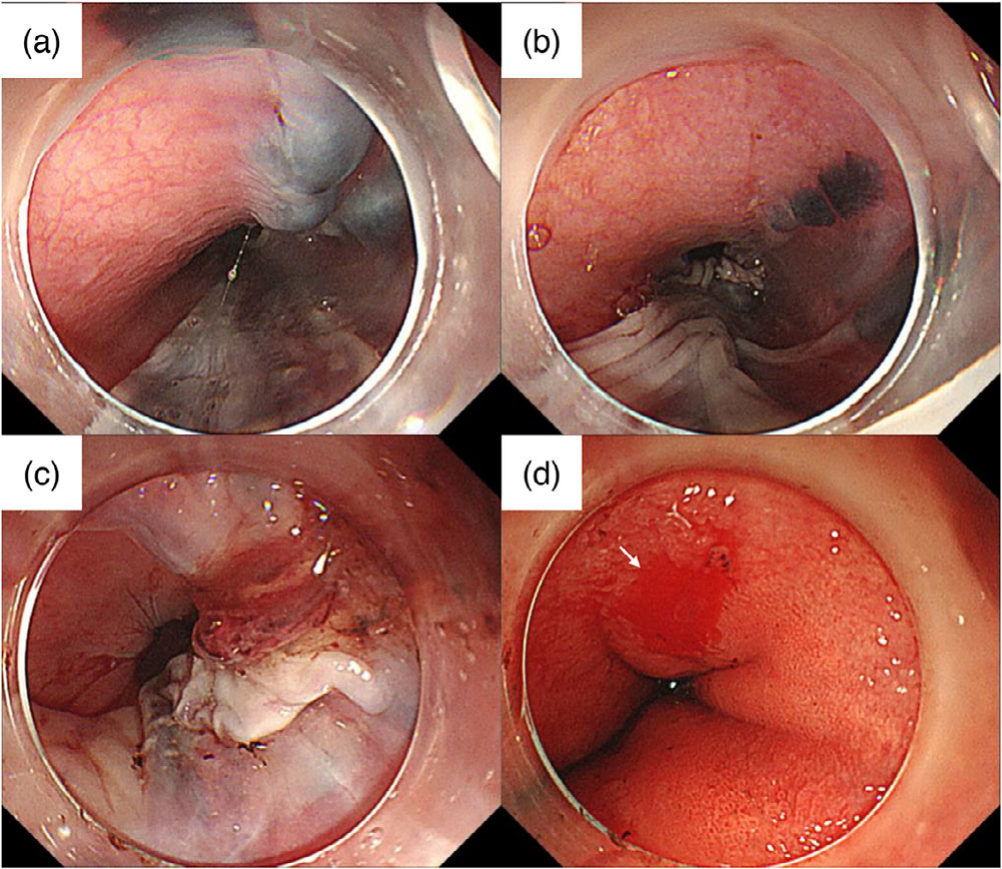
Endoscopic pictures approximately 3 h after hematemesis. (a) The esophageal submucosal hematoma was observed in the upper esophagus. (b) The esophageal submucosal hematoma largely collapsed in the middle esophagus. (c) A blood clot was observed in the right anterior wall of the lower esophagus. (d) Image showing the mucosal laceration on the lesser curvature of the gastric cardia, leading to the diagnosis of Mallory‐Weiss syndrome (white arrow).

**FIGURE 4:**
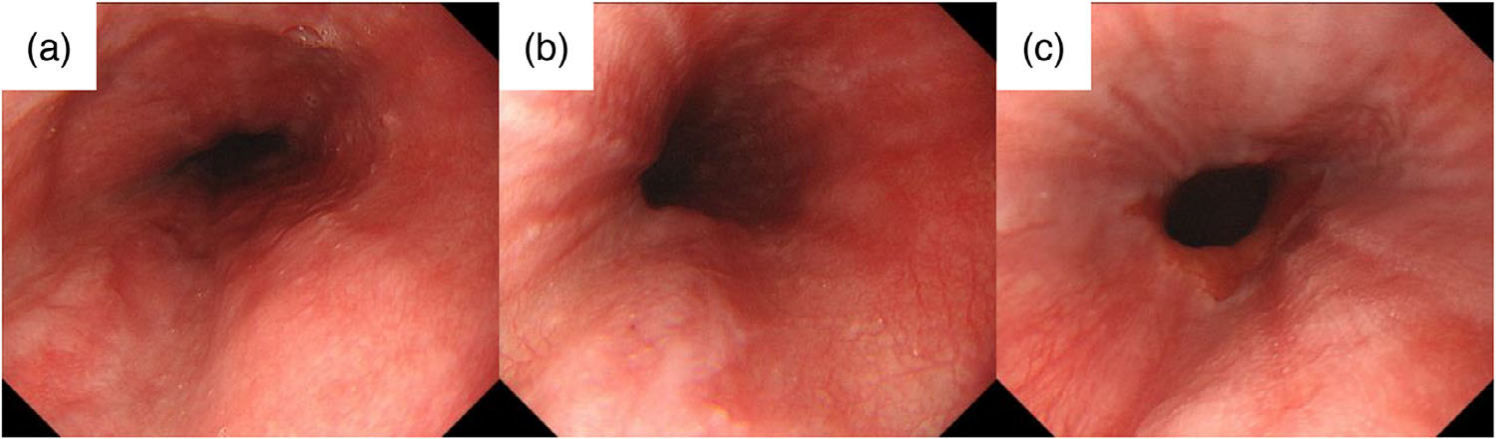
Endoscopic pictures of the esophagus on the 28th day. (a) In the upper esophagus, the esophageal submucosal hematoma was healed. (b) In the middle esophagus, the esophageal submucosal hematoma was healed. (c) In the lower esophagus, there was mucosal scarring and no esophageal stenosis.

EGD under sedation performed on the same day for further assessment and treatment revealed a purplish submucosal tumor‐like lesion extending vertically from the upper thoracic esophagus to the EGJ (Figure 3a–c). An approximately half circumferential lesion from the anterior to the posterior wall of the esophagus was largely collapsed. The right anterior wall of the lesion in the lower esophagus was covered with blood clots, suggesting a mucosal laceration. A similar mucosal laceration with adherent blood was observed on the lesser curvature of the gastric cardia (Figure 3d). Based on these endoscopic findings, the patient was diagnosed with a combination of esophageal submucosal hematoma and Mallory‐Weiss syndrome. The patient declined hospitalization, opted for conservative treatment, and was prescribed vonoprazan 20 mg for 28 days. At a follow‐up outpatient visit on day 28, the precordial pain had resolved with no subjective symptoms, and a healed hematoma was observed on follow‐up EGD (Figure 4a–c).

## DISCUSSION

To our knowledge, this is the first report of esophageal submucosal hematoma occurring during TNE, suggesting that esophageal submucosal hematomas can occur during TNE.

Esophageal submucosal hematomas are classified as either traumatic or spontaneous. Traumatic types are often associated with invasive endoscopic procedures such as endoscopic injection sclerotherapy and endoscopic retrograde cholangeopancreatography, where mechanical stress from needle punctures or large‐diameter scopes can damage the mucosa. Contrastingly, spontaneous types are linked to coagulopathy and abrupt increases in esophageal pressure due to nausea or vomiting, leading to the rupture of the submucosal esophageal vessels and subsequent hematoma formation. This case appeared to be a combination of the traumatic type, resulting from endoscopic contact, and the spontaneous type, triggered by the vomiting reflex. The patient's esophageal mucosa might have already weakened owing to regular alcohol consumption and reflux esophagitis. Therefore, even minor trauma from a less invasive, small‐caliber endoscope might have been sufficient to cause damage.

In patients with normal hemostasis, submucosal hematoma after vomiting often occurs in the lower esophagus, suggesting that the pressure from vomiting damages the mucosa and submucosal vessels.^5^ In this case, the laceration in the esophageal submucosal hematoma was located on the right anterior wall of the distal esophagus, an area susceptible to increased intra‐abdominal pressure.^6^ Hematoma formation may have initially originated in the lower esophagus, with mucosal laceration causing the hematoma to bleed and collapse thereafter. Subsequently, bleeding from the ruptured submucosal vessels in the lower esophagus is thought to have extended into the submucosa of the upper esophagus, forming a widespread esophageal submucosal hematoma.

The risk of esophageal submucosal hematoma in standard transoral endoscopy compared to TNE is not well defined, with no reported cases of standard transoral endoscopy in patients without coagulopathy. The vomiting reflex and mechanical stress are key to hematoma development, suggesting that standard endoscopy might have a higher risk than small‐caliber endoscopy because of more significant esophageal invasion and the potential to induce vomiting.

The vomiting reflex during EGD resulted from the stimulation of the base of the tongue and gastric distention due to insufflation and endoscopic contact.^7^ Factors contributing to increased EGD discomfort include young age, female sex, anxiety, smoking, and hiatal hernia presence.^8^, ^9^ Additionally, the intensity of the vomiting reflex might be linked to impaired stomach contraction, a critical factor in regulating food intake.^10^ In this patient, intense vomiting after duodenal endoscopic insertion suggests that gastric distention caused by endoscopic contact was the primary trigger. Despite having no organic disease, her significant weight loss and low body mass index (13.3), along with young age, hiatal hernia, and stress, likely exacerbated the vomiting reflex and gastric discomfort.

Although Mallory‐Weiss syndrome and esophageal submucosal hematoma share the commonality of increased intra‐abdominal pressure, the rarity of the esophageal submucosal hematoma signifies unique risk factors. This case indicates that rapid weight loss, alcohol consumption, and reflux esophagitis are risk factors. While the direct link between these factors and esophageal submucosal hematomas remains unclear, weakened esophageal tissue may predispose patients to hematoma development. In addition, despite concerns regarding delayed healing due to malnutrition, effective healing was achieved using a gastric acid inhibitor. Consequently, these factors do not significantly impede the recovery process, though they play a role in the onset of the disease.

In conclusion, this is the first report of esophageal submucosal hematoma during TNE, emphasizing the importance of esophageal submucosal hematoma and Mallory‐Weiss syndrome in the differential diagnosis of hematemesis in similar scenarios. Factors such as severe thinness, daily alcohol consumption, and reflux esophagitis in this patient may have contributed to the development of the esophageal submucosal hematoma. Further studies are necessary to improve the safety of endoscopic examinations and identify the exact risk factors.

## CONFLICT OF INTEREST STATEMENT

None.

## References

[1] Hamashima C , Fukao A . Quality assurance manual of endoscopic screening for gastric cancer in Japanese communities. Jpn J Clin Oncol 2016; 46: 1053–1061. 27589938 10.1093/jjco/hyw106

[2] Alexandridis E , Inglis S , McAvoy NC *et al.* Randomised clinical study: Comparison of acceptability, patient tolerance, cardiac stress and endoscopic views in transnasal and transoral endoscopy under local anaesthetic. Aliment Pharmacol Ther 2014; 40: 467–476. 25039412 10.1111/apt.12866

[3] Salomez D , Ponette E , Van Steenbergen W . Intramural hematoma of the esophagus after variceal sclerotherapy. Endoscopy 1991; 23: 299–301. 1743137 10.1055/s-2007-1010694

[4] Zippi M , Hong W , Traversa G . Intramural hematoma of the esophagus: An unusual complication of endoscopic retrograde cholangiopancreatography. Turk J Gastroenterol 2016; 27: 560–561. 27852551 10.5152/tjg.2016.16417

[5] Shay SS , Berendson RA , Johnson LF . Esophageal hematoma. Four new cases, a review, and proposed etiology. Dig Dis Sci 1981; 26: 1019–1024. 7028429 10.1007/BF01314765

[6] Kinoshita Y , Furuta K , Adachi K , Amano Y . Asymmetrical circumferential distribution of esophagogastric junctional lesions: Anatomical and physiological considerations. J Gastroenterol 2009; 44: 812–818. 19526190 10.1007/s00535-009-0092-0

[7] Enomoto S , Watanabe M , Yoshida T *et al.* Relationship between vomiting reflex during esophagogastroduodenoscopy and dyspepsia symptoms. Dig Endosc 2012; 24: 325–330. 22925284 10.1111/j.1443-1661.2012.01241.x

[8] Majima K , Shimamoto T , Muraki Y . Causative factors of discomfort in esophagogastroduodenoscopy: A large‐scale cross‐sectional study. World J Gastrointest Endosc 2020; 12: 128–137. 32341749 10.4253/wjge.v12.i4.128 PMC7177206

[9] Campo R , Brullet E , Montserrat A *et al.* Identification of factors that influence tolerance of upper gastrointestinal endoscopy. Eur J Gastroenterol Hepatol 1999; 11: 201–204. 10102233 10.1097/00042737-199902000-00023

[10] Janssen P , Verschueren S , Tack J . Intragastric pressure as a determinant of food intake. Neurogastroenterol Motil 2012; 24: 612–615.e267‐8. 22519421 10.1111/j.1365-2982.2012.01911.x

